# The Effects of COVID-19 on Orthopaedic Surgery Training Programs in the United States

**DOI:** 10.5435/JAAOSGlobal-D-22-00253

**Published:** 2023-05-02

**Authors:** Shivan N. Chokshi, Tsola A. Efejuku, Jie Chen, Daniel C. Jupiter, Jeremy S. Somerson, Vinod K. Panchbhavi

**Affiliations:** From the John Sealy School of Medicine (Mr. Chokshi and Mr. Efejuku), Department of Orthopaedic Surgery and Rehabilitation (Dr. Chen, Dr. Jupiter, Dr. Somerson, and Dr. Panchbhavi), Department of Preventive Medicine and Population Health (Dr. Jupiter), The University of Texas Medical Branch, Galveston, TX.

## Abstract

This study investigates the effects of the COVID-19 pandemic on medical education, research opportunities, and mental health in orthopaedic surgical training programs.

**Methods:**

A survey was sent to the 177 Electronic Residency Application Service–participating orthopaedic surgery training programs. The survey contained 26 questions covering demographics, examinations, research, academic activities, work settings, mental health, and educational communication. Participants were asked to assess their difficulty in performing activities relative to COVID-19.

**Results:**

One hundred twenty-two responses were used for data analysis. Difficulties were experienced in collaborating with others (49%), learning through online web platforms (49%), maintaining the attention span of others through online web platforms (75%), and in gaining knowledge as a presenter or participating through online web platforms (56%). Eighty percent reported that managing time to study was the same or easier. There was no reported change in difficulty for performing activities in the clinic, emergency department, or operating room. Most respondents reported greater difficulty in socializing with others (74%), participating in social activities with coresidents (82%), and seeing their family (66%). Coronavirus disease 2019 has had a significant effect on the socialization of orthopaedic surgery trainees.

**Discussion:**

Clinical exposure and engagement were marginally affected for most respondents, whereas academic and research activities were more greatly affected by the transition from in-person to online web platforms. These conclusions merit investigation of support systems for trainees and evaluating best practices moving forward.

The effect of the coronavirus disease 2019 (COVID-19) pandemic has forced institutions and individuals to rapidly adjust daily operations, procedures, and processes. In response to the pandemic, surgical programs across the country implemented shifts to online didactics, distancing protocols, and stricter guidelines for surgical opportunities.^1^ These changes introduced new challenges for residents already regularly experiencing high levels of stress due to long hours, strenuous clinical workloads, and limited professional and personal control.^2^ While there is an established body of literature detailing specific effects of COVID-19 on surgical residency programs, a majority of it examines other countries such as India, Algeria, and Pakistan, or focuses on general surgery residents.^1,3-5^

Orthopaedic residency programs have continued to grow in popularity, particularly in the last decade. National Residency Match Program data have demonstrated increases in both applications and admissions to US medical school orthopaedic surgery residency programs. Given this rise in popularity, we sought to better understand how the COVID-19 pandemic has affected the academics, research, and mental health of residents in orthopaedic surgical residency and fellowship programs in the United States. Our objectives were to determine how residents and fellows are coping with this transition, what limitations trainees are facing, and which aspects of these programs could be improved to support their trainees' education.

## Methods

The UTMB Health Institutional Review Board reviewed this study and deemed it exempt according to our institution's policies. A cross-sectional multiple-choice online survey using Microsoft Forms (Microsoft) was sent to 177 orthopaedic surgery training programs participating in the Electronic Residency Application Service, targeting program coordinators and directors to disseminate the survey to postgraduate year 1 through 5 residents and first- and second-year fellows. The total number of active orthopaedic residents and fellows at the time the survey was sent out was 4,766.^6^ One reminder e-mail was sent 4 weeks after the first e-mail.

The survey contained 26 questions divided into 5 sections (Supplemental Table 1, http://links.lww.com/JG9/A277). Survey questions were adapted from a 2020 analysis of the effects of COVID-19 on orthopaedic surgery residency programs in North India.^5^ In Part I of the survey, participants were asked to provide general information, including the location of their institution, year designation, age, sex, COVID-19 exposure, testing, and quarantine history. Part II, Academic Activities, was subdivided into 3 subsections: examinations, research, and general activities. Part III, Clinical Activities, was subdivided into 3 subsections: working in the operating room, emergency department and clinical departments, and inpatient wards. Part IV, Mental Health and Communication, was subdivided into 2 subsections: mental health effects and educational communication. Part V, Overall Impact, assessed the use of technological simulations and limitations on space. An optional free-response question for any additional comments was placed at the end of each section to allow respondents the space to elaborate on their experiences.

Participants were asked to reflect on the current difficulty of doing specific activities relative to a time before COVID-19. All participants were asked to provide a single answer for all the questions within the academic activities, clinical activities, and mental health and communication sections, choosing between very easy, easy, same, difficult, and very difficult. An answer choice of N/A was also offered in these sections for questions for which participants did not have a suitable answer. For the Overall Impact section, answer choices of yes or no were provided. Collected data were exported to a database for descriptive analysis.

## Results

**Figure 1:**
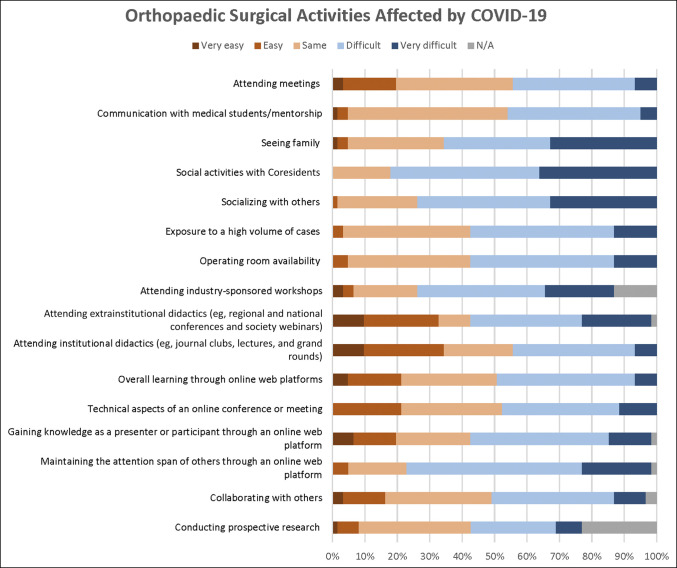
Graphical overview of orthopaedic surgical activities that were reported as difficult or very difficult by most respondents.

**Figure 2:**
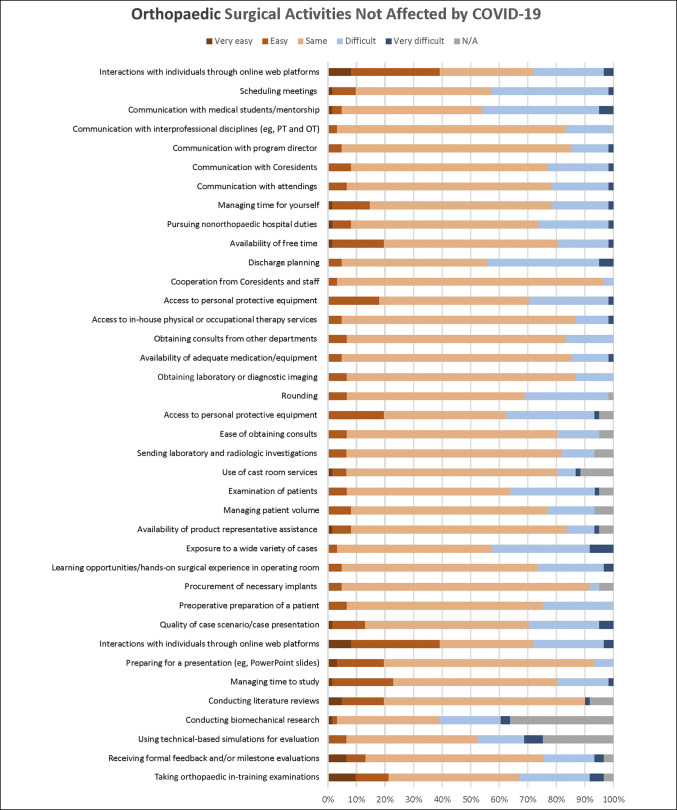
Graphical overview of orthopaedic surgical activities that were reported as same, easy, or very easy by most respondents.

A total of 122 completed questionnaires were received from 113 residents (93%) and 9 fellows (7%). The mean age of participants was 31 years. Most residents were postgraduate year 4 (26%). Most respondents were male (72%), and most participants (89%) reported treating COVID-19-positive patients at some point in time. However, only 10 (8%) reported testing positive for COVID-19 (see Supplemental Table 2, http://links.lww.com/JG9/A278, for a summary of results from the survey). Geographically, the most represented state in the survey was Texas, with 28 responses (23%). Substantial numbers of responses were also seen from Pennsylvania (6), California (5), and Ohio (4). Figure 1 displays the activities that survey respondents felt were negatively affected by COVID-19. Figure 2 displays activities that respondents felt were not negatively affected by COVID-19.

### Academic Activities

Nearly half (45%) of the respondents found conducting prospective research more difficult than previously. A smaller percentage of the respondents found conducting biochemical research more difficult (39%). Retrospective research and literature reviews were least affected by COVID-19; 101 respondents (83%) did not report an increase in the difficulty of conducting retrospective research, and 110 respondents (90%) did not report an increase in performing literature reviews. Importantly, however, nearly half of respondents (49%) reported that collaborating with others in a research context was more difficult.

Overall, examination-related activities were not significantly affected by COVID-19. Fifty-six respondents (46%) reported no change in the difficulty of taking orthopaedic in-training examinations, and 16 (21%) found examinations to be easier. Similarly, a majority (62%) of respondents reported no change in the difficulty of receiving formal feedback and/or milestone evaluations.

Most of the residents did not experience greater difficulty in managing time to study (80%), preparing for a presentation (93%), or interacting with individuals through online web platforms (72%). However, most respondents expressed increased difficulty in maintaining the attention span of others (75%) and gaining knowledge as a presenter or participant (56%) through online web platforms. Although a minority of respondents found a decrease in the quality of case presentations (30%), nearly half found it more difficult overall to learn through web platforms (49%).

COVID-19 had effects on attending group activities. Many respondents (44%) expressed greater difficulty attending institutional didactics, such as lectures and grand rounds. A majority found it more difficult to attend extrainstitutional didactics, such as regional/national conferences (56%) and industry-sponsored workshops (61%). Additional comments received in this section noted a preference for “in-person over web-based learning experiences [due to] easier interaction with presenter/audience.” In addition, one respondent commented that “[their] program did not move to virtual didactics during COVID.”

### Clinical Activities

Respondents reported that personal protective equipment (PPE) was more difficult to obtain in the operating room (43%), the emergency department (33%), and the inpatient department (30%). Although this was not a challenge expressed by most respondents, it is still significant, given the importance of PPE to disease prevention during the pandemic. It was noted that although there was not a significantly increased difficulty with learning opportunities and hands-on surgical experience (26%), respondents still reported greater difficulty with operating room availability (57%), exposure to a high volume of cases (57%), and exposure to a wide variety of cases (43%). One respondent noted in a comment that the biggest limitations were seen with “surgical skills training and anatomy training, which [were] non-existent for the past year and a half.”

One-third of respondents (33%) reported greater difficulty in examining patients in the emergency department or a clinical setting. However, most residents and fellows did not experience greater difficulty in managing patient volume (77%) or obtaining consults (80%).

In inpatient settings, a minority of residents reported increased difficulty with rounding (30%) and discharge planning (44%). Specific comments were offered, noting that “discharge to rehab facility or nursing homes [took] twice or 3 times as long to be approved/accepted. This caused significant issues with hospital capacity, and elective cases were cancelled due to no open beds.” This frustration was echoed by another respondent, who noted that the difficulties in discharge planning led to “[utilization] of bed space and resources.”

### Mental Health

Most residents and fellows found socializing with others (74%), social activities with coresidents (82%), and seeing family (66%) to be more difficult after the start of the COVID-19 pandemic. A smaller proportion reported decreased free time (20%) and greater difficulty pursuing nonorthopaedic hospital duties (26%) and managing time for themselves (21%). Last, over one-third (39%) of respondents experienced an increase in anxiety while working due to fear of contracting COVID-19.

No significant increased difficulty in communicating with attending residents (21%), coresidents (23%), or program directors (15%) was reported. However, nearly half of all respondents found it more difficult to communicate with medical students and provide mentorship (46%), schedule meetings (43%), and attend meetings (44%).

### General Effect

Overall, 44 respondents (36%) reported an increase in the use of technological simulations at their institution because of COVID-19. Of these, 26 (59%) found the increased use of simulations beneficial to their educational experience. Sixty residents (49%) indicated that their institution implemented a limitation on the number of people that could scrub into the operating room at a given time, and exactly half of those (50%) thought that this limitation negatively affected their learning opportunities. An individual comment noted that “anatomy labs, industry-sponsored courses, [and] AO/AAOS courses have not been taking place. This is having a significant effect for junior residents who have never experienced these events and may not be doing any in-person skill training due to COVID.”

## Discussion

Overall, the greatest difficulties were seen with research collaboration, maintaining engagement through online platforms, attending extrainstitutional didactics, seeing family, providing medical student mentorship, and scheduling/attending meetings (Figure 1). Although the use of simulations was not broadly expanded, most of those who reported such an increase found it beneficial. Restrictions on capacity in the operating room also negatively affected half of those to whom it applied.

The results of the survey align with the experiences of residents in other countries. In India, residents reported difficulty maintaining the attention span of an audience through online web platforms, socializing with others, and obtaining PPE.^5^ Likewise, orthopaedic residents in South Korea were dissatisfied with online teaching methods, increased isolation, decreased quality of life, and decreased time in the operating room.^7^ Similar effects were also seen within programs in Karachi, Pakistan, and British Columbia.^8,9^

The COVID-19 pandemic was an opportunity to learn more about resident education and specific modes of optimal learning. Most residents did not find learning through web platforms more challenging, and nearly a quarter reported that online learning was easier (Figure 2). Given that maintaining the attention span of others was the most significant challenge, although there was a minimal negative effect on the quality of case presentations or the ease of preparing for presentations, it is important to explore a continued use of online platforms while expanding strategies to improve engagement. For instance, the implementation of a weekly surgical game-show-style quiz program during general surgery education conferences was correlated with improved test scores.^10^ The Emory University Department of Orthopaedics reported on a 2-team system in which a group of remotely working residents participated in daily faculty-directed videoconferences, didactics, and quality improvement projects.^11^ At Harvard University, the program prepared physical models and delivered practice equipment to the homes of trainees as part of their intern-year surgical skills training.^12^ These types of interactive modes of learning have the ability to build on the benefits of web-based education, while attenuating the reported drawbacks. The results of the survey have also uncovered the undeniable importance of in-person institutional and extrainstitutional events to improve resident education and overall experience.

COVID-19 is highly contagious, which augments the concerns expressed by respondents in obtaining PPE in various settings. Particularly in the emergency department, where COVID-19 transmission is more likely, almost a third of respondents reported more difficulty in obtaining PPE. This figure was even higher when asked in the context of the operating room. These challenges reflect a national shortage in PPE seen in dozens of hospitals due to spiking demands and disruptions of global supply chains.^13^ Similar findings have been reported in other countries as well; in a survey of orthopaedic and trauma surgeons in Germany, only 70% of respondents felt that adequate PPE was supplied.^14^ This lack of access to PPE may represent a contributing factor to the increase in anxiety while working, as expressed by nearly 40% of residents.

The mental health effects on orthopaedic surgery residents and fellows align closely with what has been observed more broadly. Recent surveys of healthcare providers have demonstrated a significant increase in anxiety and depression when treating patients with COVID-19.^15,16,17,18^ Before the start of the pandemic, many graduate medical training programs were implementing policy changes centered around improving resident trainee mental health. A study conducted at Stanford University Hospital provided residents with a wellness app that encouraged mindfulness, stress management, and meditation. Most participants displayed improvement in their baseline positive affect and mindfulness scores.^19^ Similarly, a training program in South Carolina implemented a voluntary wellness initiative for residents aimed at combating anxiety, depression, poor quality of life, and sleepiness. For 1 year, participants attended weekly group workouts and biweekly lectures on sleep hygiene and mental health. Residents reported an increase in baseline anxiety, quality-of-life, and sleepiness scores, and overall favorable perception of the program.^20^

The use of simulations incorporating new technologies and high-fidelity recreation of procedures in surgical programs constrained by staffing limitations and COVID-19 protocols can benefit residents, fellows, and surgeons, while decreasing hospital costs and improving patient outcomes.^21^ Twenty-two respondents (36%) reported an increase in the use of technological simulations, and most of those (59%) found the increase beneficial to their educational experience. The relatively low rate of increased simulation use in the wake of COVID-19 is both an interesting finding and an opportunity for institutions to understand their benefit and consider their value in the future.

With COVID-19 variants spreading across the globe, the results of our survey are even more relevant as we move toward a different phase of the pandemic. As of late 2021, one class of orthopaedic surgery residents have already started their postgraduate training amid COVID-19 restrictions and regulations, and another has just begun. The possible long-term repercussions of this prolonged shift in training curricula are unknown, but we hope that results from this study can be used by orthopaedic surgery training programs to address trainee concerns about research collaboration, maintaining engagement, attending extrainstitutional didactics, seeing family, and providing mentorship, as well as greater integration of simulations to supplement surgical skills training and operating room exposure.

The most significant limitation to this study is the poor response rate. Contact information for all orthopaedic surgery residents and fellows is not publicly available, so we relied on program coordinators and/or program directors to forward the survey to their trainees. Thus, although there are a total of 4,766 orthopaedic surgery residents and fellows in the United States, we cannot be sure that the survey was actually forwarded from program coordinators and thus many may not have had an opportunity to complete the survey at all. As with any survey, response bias may influence the results. Those who were more significantly affected by the ramifications of COVID-19 are more likely to respond to the survey. In addition, baseline data regarding opinions toward the survey questions before COVID-19 were not obtained. However, we are still able to make conclusions because the survey was specifically designed to elicit opinions in a relative manner to determine the effect of COVID-19 based on each individual's baseline.

COVID-19 has had a significant effect on orthopaedic surgery trainees. The effects seen are 3-fold. First, difficulties were seen with activities that required in-person interactions, such as research collaboration, educational engagement, extrainstitutional didactics, rounding and discharge planning, and attending meetings. Second, difficulties were reported regarding global disturbances as a result of COVID-19—namely the lack of PPE available in critical situations. Third, difficulties were seen due to a lack of social support, from being unable to engage in social activities and see family.

The use of simulations was not broadly expanded, but a generally positive impression of simulations signifies an opportunity for additional implementation more broadly. A more comprehensive use of technological simulations across the country may alleviate the difficulties experienced with capacity limitations implemented in the operating room. These conclusions merit further evaluation of support systems for trainees and consideration of best practices moving forward.
